# Meta-analysis of the relationship between university students' anxiety and academic performance during the coronavirus disease 2019 pandemic

**DOI:** 10.3389/fpsyg.2023.1018558

**Published:** 2023-03-14

**Authors:** Yuxi Tang, Weiguang He

**Affiliations:** College of Social Sciences, Shenzhen University, Shenzhen, China

**Keywords:** COVID-19 pandemic, student anxiety, academic performance, university student, meta-analysis

## Abstract

**Introduction:**

The COVID-19 pandemic has had a profound impact on the mental health and academic performance of university students worldwide. Anxiety is one of the most commonly reported mental health issues among this population, but its relationship with academic performance during the pandemic has not been fully explored.

**Methods:**

A meta-analysis was conducted following the PRISMA-P guidelines to synthesize existing research on the relationship between anxiety and academic performance in university students during the COVID-19 pandemic. Four databases were searched (PsycINFO, Web of Science, PubMed, and Scopus) for articles published between December 2019 and June 2022, and studies from five different countries were included in the analysis. A heterogeneity test was performed, and a fixed-effects model was used to calculate the main results.

**Results:**

The meta-analysis revealed a negative correlation between university students' anxiety and academic performance (*r* = −0.211, *k* = 5, *N* = 1,205). Subgroup analysis found no significant regulatory effects for the year of publication, country development level, student type, or anxiety type. The results suggest that negative emotions induced by the pandemic are the most significant factor linking anxiety to poor academic performance.

**Discussion:**

During pandemics with severe global consequences, such as COVID-19, interventions against and for the prevention of university students' negative emotions are important ways to improve university students' mental health and academic achievement.

## 1. Introduction

The continued prevalence of coronavirus disease 2019 (COVID-19) has not only caused many casualties worldwide but also seriously affected the mental health of university students from different countries by engendering anxiety among this group (Islam S., et al., 2020; Huarcaya-Victoria et al., 2021; Visser and Law-van Wyk, 2021). The anxiety level of university students worldwide has generally increased, which has harmed their academic performance. Understanding the relationship between the anxiety and academic performance of university students and the factors that influence this relationship during the COVID-19 pandemic would be helpful for educational managers to formulate appropriate policies and intervention methods. However, to date, no meta-analysis has assessed this issue. Meta-analysis can help people comprehensively examine and generalize the relationship between academic performance and anxiety and obtain more reliable conclusions. A comprehensive meta-analysis is needed to analyze the relationship between anxiety and academic performance of college students in the context of the pandemic. When educational administrators have a clear understanding of the enormous negative consequences of college students' anxiety, they are more likely to develop systematic ways to intervene in college students' anxiety by protecting college students' mental health and providing them with more social support.

Anxiety is usually defined as a mental disorder (Spitzer et al., 2006) and an emotional state that pertains to coping with possible negative future events (McNaughton, 2018). Many studies have shown that university students are prone to anxiety disorders (Sakin Ozen et al., 2010; Walters et al., 2018; Wang et al., 2020; Li et al., 2022). Previous studies have suggested that anxiety among university students may be engendered by an inability to adapt to new situations, worry about future uncertainty, difficulty in solving problems, and negative life experiences (Vitasari et al., 2010; Arbona et al., 2021; Sustarsic and Zhang, 2022). There are many specific drivers of university students' anxiety, such as learning (Islam M. A., et al., 2020), exams (Fernández-Castillo and Caurcel, 2015; Hamzah et al., 2018), and problematic Internet use (Lozano Blasco et al., 2020). Academic stress is the main cause of anxiety among university students due to their heavy academic load (Zhang et al., 2022). In addition, social and economic pressures may cause anxiety (Jones et al., 2018). The reasons for university students' anxiety also include the transformation of how courses are delivered (i.e., from in-person to remote), increased academic burden, lack of technical literacy, economic difficulties, health concerns, and reduced social interactions (Deng et al., 2021).

Academic performance is defined as the extent to which students have mastered course knowledge and skills, the ability to complete academic tasks, and the overall academic results achieved (Adediwura and Tayo, 2007; Richardson et al., 2012; Talsma et al., 2018). The factors and mechanisms that influence college students' academic performance have been a topic of interest, especially in light of the increased academic challenges faced by college students during the pandemic. College students' academic achievement is influenced by numerous components, including cognition (Singh et al., 2016), psychology (MacCann et al., 2020), and social environment (Doleck and Lajoie, 2018). Among these, mental health has a greater impact on the academic performance of college students.

Past studies have suggested that university students' academic and non-academic anxiety harm learning achievements (Leppavirta, 2011; Adeoye-Agboola and Evans, 2015; Zhang et al., 2019; Liu and Xu, 2021). However, a few studies have also found that students' test of anxiety is not significantly correlated with academic performance (Karjanto and Yong, 2013), which implies that research in this area needs to be further developed. Previous meta-analyses have revealed correlations of −0.21 (Seipp, 1991), −0.28 (Erzen, 2017), and −0.06 (Brumariu et al., 2022) between students' anxiety and overall academic performance. However, the effect sizes reported in previous studies are inconsistent. In Brumariu et al. (2022), the effect sizes for adult students were quite small, while the effect sizes reported by Seipp (1991) and Erzen (2017) were between small and moderate. Thus, it is necessary to conduct meta-analyses that use rigorous methods to determine the true effect sizes. Seipp (1991) did not distinguish between student groups. Furthermore, although Erzen (2017) analyzed college students as a subgroup, however, the study did not distinguish between undergraduate and graduate students. Brumariu et al. (2022) considered the age of students as an important basis for subgroup analysis but did not distinguish between undergraduate and graduate students. Because the learning situation of students in different education and learning stages is quite different, it is necessary to select college students separately for meta-analysis.

In addition, previous meta-analyses did not consider the special context of the pandemic. In the current situation, which is exemplified by the repeated impact of the pandemic, it is especially urgent to expand the meta-analyses in this area. Therefore, this meta-analysis addresses the following two issues: (1) the overall correlation between university students' anxiety and academic performance during the COVID-19 pandemic and (2) variables regulating the relationship between university students' anxiety and academic performance during the COVID-19 pandemic.

## 2. Methods

This study followed the PRISMA-P guidelines (Moher et al., 2015). A detailed checklist is presented in Supplementary Table 1.

### 2.1. Literature search

When conducting a meta-analysis, it is necessary to select an appropriate research database (Gusenbauer and Haddaway, 2020). Accordingly, we primarily selected PsycINFO, Web of Science, PubMed, and Scopus as the main search databases and used Google Scholar and Semantic Scholar databases for the supplementary search. We also manually searched the references of the preliminary selected articles to find supplementary articles that met the selection criteria. After discussion, the two researchers reached an agreement on the retrieval strategy and carried out independent literature searches. The search terms used were “college student” or “university student,” “undergraduate” or “graduate,” “COVID-19 pandemic,” “COVID-19,” or “2019 coronavirus,” “anxiety,” “worry,” or “fear,” “academic performance” or “academic achievement,” and “test scores” or “learning achievement.” Since the COVID-19 pandemic began in December 2019, the publication date was limited to between 1 December 2019 and 30 June 2022. More details regarding the literature search strategy are shown in Supplementary Table 2. After reading the title, abstract, and body of the article, the two researchers included articles that they believed met the selection criteria. Duplicate articles were removed. The two researchers discussed the different articles until they reached an agreement regarding the inclusion of the article. If the two researchers could not reach an agreement, a third reviewer was invited to participate in the discussion and determine the preliminary selected articles.

### 2.2. Inclusion and exclusion criteria

The two researchers primarily selected articles written in English that studied the relationship between university students' anxiety and their academic performance during the COVID-19 pandemic. Specifically, research articles that included university students as participants were selected. There were no specific requirements regarding the research design, but the selected research needed to be within the context of the COVID-19 pandemic, contain assessments of university students' anxiety and academic performance, and analyze the correlation between university students' anxiety and academic performance. Anxiety could be assessed through physiological measurement or self-report. Academic performance could be assessed as actual measured academic performance or as self-reported performance. Articles that did not meet these standards were discarded.

### 2.3. Data extraction

The two researchers first discussed the data available in the research articles. After a discussion, they agreed that the following information should be collected: author, year of publication, country, research design, student type, the proportion of female students, average age, student major, sample size, anxiety assessment tools used, methods of evaluating academic performance, effect size, and research quality. The two researchers first extracted relevant data independently and then engaged in a discussion to reach an agreement on any divergent content.

### 2.4. Quality assessment

Previous researchers have used the Joanna Briggs Institute Methodological Quality Questionnaire (MQQ) framework (Acosta et al., 2020) to evaluate the methodological quality of the selected articles. This method quality checklist provides researchers with a basis for critical evaluation in terms of nine dimensions: theoretical definition, operational definition, research design, sample design, sample description, data credibility, data analysis, practical impact, and policy impact. The MQQ contains nine questions, each of which receives a score of 0, 1, or 3, such that the overall maximum score of the questionnaire is 27. In this evaluation standard, since each question includes a two-step review, a score of 0 or 1 is assigned in the first step review. Only after obtaining a score of 1 in the first step review is the second step review conducted. The second review assigns a score of 0 or 2 so that the final possible review score for each question cannot include the value 2. We scored the methodological quality of the preliminary selected articles according to the MQQ standard. A total score of 19–27 points indicated high quality; 9–18 points, medium quality; and 0–8 points, low quality. Low-quality research articles were eliminated from the analysis.

### 2.5. Data analysis

This meta-analysis used Pearson's *r* to measure the correlation between university students' anxiety and academic performance. If Pearson's *r* was reported directly in a given study, no conversion was necessary. If other measures of effect size were reported instead, the method provided by Borenstein (2009) was used to uniformly convert other types of effect sizes into *r* values. It was necessary to analyze the heterogeneity among the included studies; *I*^2^ was used for this purpose (Huedo-Medina et al., 2006). When calculating the total effect, researchers need to choose whether to use a fixed-effect or a random-effect design according to the heterogeneity among studies. In addition, publication bias was assessed, and a sensitivity analysis was conducted (Copas and Shi, 2000). To assess publication bias, a funnel plot was inspected for symmetry (Sterne et al., 2011) in combination with Egger's expression test (Lin and Chu, 2018). In the sensitivity analysis, the selected studies were excluded individually, and the changes in the combined effect values before and after exclusion were observed to judge whether the results were robust. Through a subgroup analysis, variables that potentially affected the overall effect were further assessed (Borenstein and Higgins, 2013).

## 3. Results

### 3.1. Literature search results

**Figure 1:**
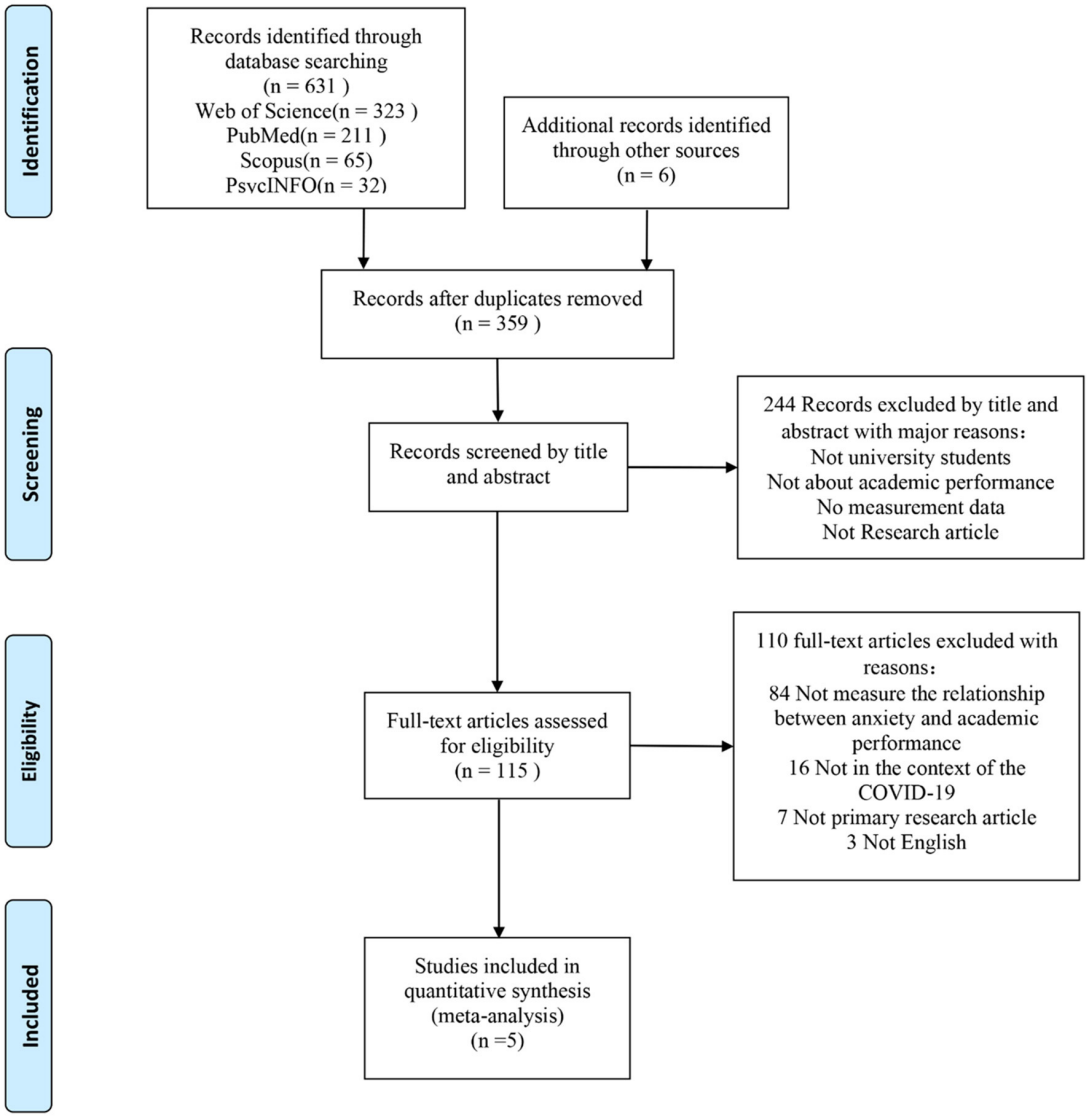
Flow diagram of studies included and excluded. From Moher et al. (2015).

We first screened the 637 articles that met the criteria independently and manually excluded 278 duplicate articles. We further read the title and abstract of the remaining 359 articles and excluded 244 articles that did not meet the selection criteria of this literature review. We read the full text of the remaining 115 articles, finally excluding 110 articles that did not meet the selection criteria. Finally, we reached a consensus that five articles should be used in the meta-analysis. Detailed information on the studies included in the meta-analysis is provided in the Supplementary material. Figure 1 shows the study inclusion and exclusion processes.

### 3.2. Research characteristics

We separately coded the included studies and used percent agreement to assess interrater reliability. The coding consistency percentages of the two researchers on the national economic development level, research type, research methodology quality, and other items were all >90%. We discussed and reached an agreement on any codes that exhibited differences. The correlation coefficient between university students' anxiety and academic performance ranged from −0.294 to −0.120. These studies were carried out in five countries, namely the United Kingdom, Indonesia, South Korea, India, and Malaysia. The years of publication of the articles were concentrated in 2021 and 2022. All of the studies were cross-sectional. Among the five studies, three recruited undergraduate students, one assessed graduate and postgraduate students, and one considered postgraduate students. The proportion of female students in the five studies ranged from 50 to 69.2%. The average age of participants ranged from 23.7 to 44.4 years. The sample sizes ranged from 100 to 436, and the total sample size was 1,205. Two of the five studies reported the academic anxiety level of university students, where one reported the test anxiety of university students, one reported the social evaluative anxiety of university students regarding COVID-19, and one reported the economic anxiety of university students. Three of the five studies used university examination results as a measure of academic performance, and two used a learning-output-related scale. Table 1 shows the main characteristics of the included studies.

**Table 1:** Characteristics of the included studies.

**References**	**Country**	**Research design**	**Student type**	**Proportion of female students**	**Average age**	**Student major**	**Sample size**	**Anxiety type**	**Anxiety assessment**	**Academic performance evaluation methods**	**Effect size *r***
Chattopadhyay and Sahoo (2022)	India	Cross-sectional	Graduate, Postgraduate	50.0%	/	Multiple majors	100	Test anxiety	Self-developed scale of test anxiety	Exam score	−0.276
Di Malta et al. (2022)	United Kingdom	Cross-sectional	Undergraduate	69.2%	44.4	Arts and social sciences	208	Generalized anxiety	Generalized anxiety disorder scale 7	Mean module scores	−0.190
Fadhila and Hernawan (2021)	Indonesia	Cross-sectional	Postgraduate	/	/	Medical	151	Generalized anxiety	Depression anxiety and stress scale 42	Exam score	−0.294
Kim and Park (2021)	South Korea	Cross-sectional	Undergraduate	86.5%	23.9	Nursing	310	Social-evaluative anxiety related to COVID-19	Social Avoidance and distress scale	Learning outcome scale	−0.120
Noman et al. (2021)	Malaysia	Cross-sectional	Undergraduate	56.9%	23.7	/	436	Financial anxiety	Financial anxiety scale	Anticipated academic performance scale	−0.239

*“/” denotes not reported*.

### 3.3. Research quality assessment

Five studies met the research quality requirements of this literature review and were included in the meta-analysis. We evaluated the five studies according to the MQQ, where four studies were rated as high-quality and one as medium-quality. Overall, the quality of the five studies met the inclusion criterion, with an average MQQ score of 21. Table 2 shows the methodological quality evaluation results of the study.

**Table 2:** Methodological quality of the included studies.

**References**	**Theoretical or conceptual definition**	**Operationaldefinition**	**Research design**	**Sampling design**	**Sample**	**Validity/Reliability or trustworthiness/ credibility evidence**	**Data analysis**	**Implications for practitioners**	**Implications for policy**	**Total methodological quality score**	**Research quality**
Chattopadhyay and Sahoo (2022)	3	3	3	3	1	3	3	1	1	21	High
Di Malta et al. (2022)	1	3	3	3	3	3	3	3	1	23	High
Fadhila and Hernawan (2021)	1	3	3	3	1	1	3	1	1	17	Moderate
Kim and Park (2021)	1	3	3	3	3	1	3	3	1	21	High
Noman et al. (2021)	3	3	3	3	1	3	3	1	3	23	High

### 3.4. Mean correlation and heterogeneity test

**Figure 2:**
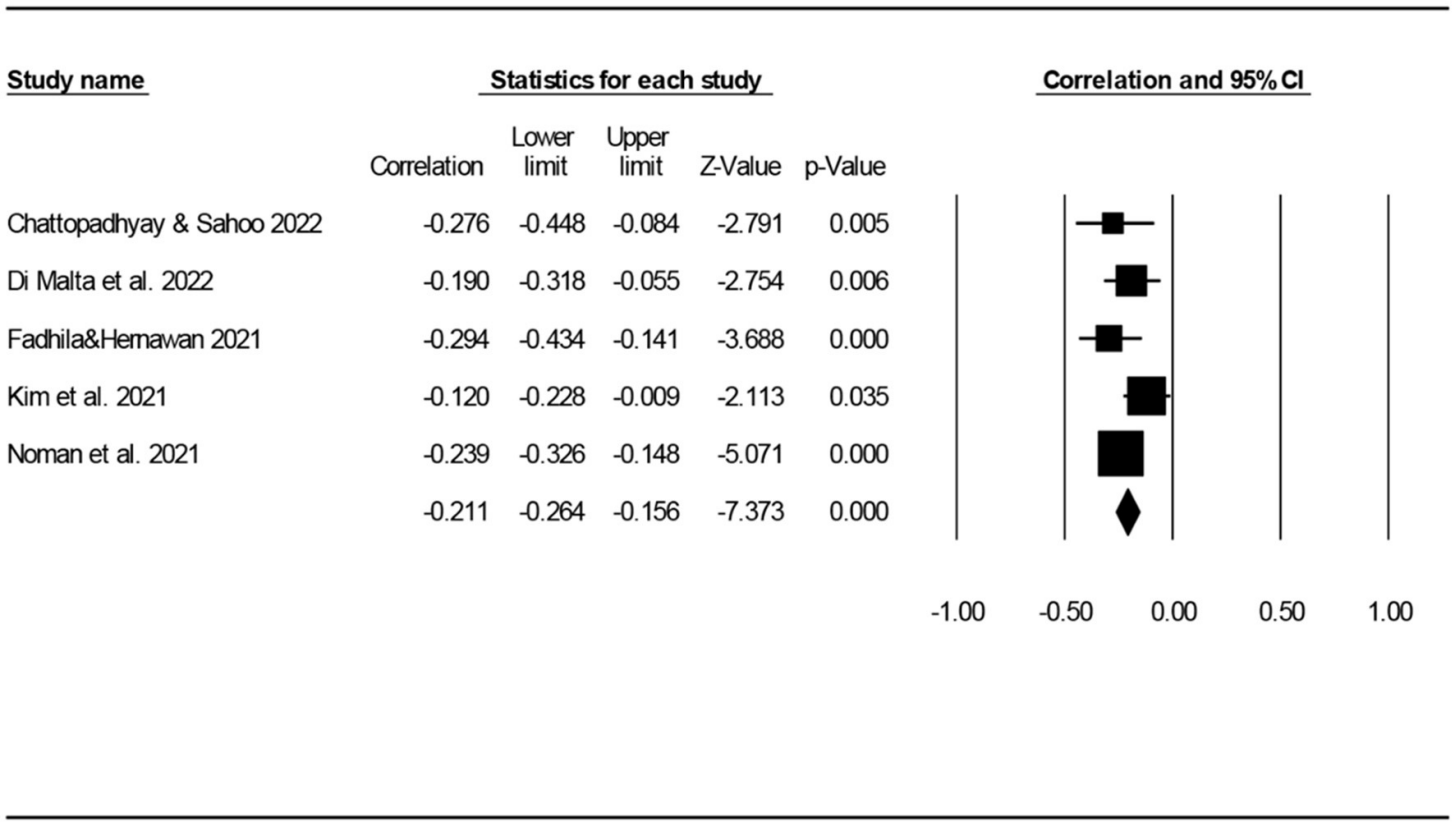
Forest plot of the relationship between the anxiety and academic performance of university students during the COVID-19 pandemic.

The correlation coefficient between university students' anxiety and academic performance in these five studies ranged from −0.294 to −0.120. Through the meta-analysis of these five studies using a fixed-effects model, the average correlation coefficient between university students' anxiety and academic performance during the COVID-19 pandemic was determined to be −0.211 (95% CI: −0.264, −0.156, *p* < 0.001). There was a low degree of heterogeneity among the five studies (*I*^2^ = 16.676%, *p* = 0.308). Figure 2 shows forest plots of the relationship between the anxiety and academic performance of university students during the COVID-19 pandemic.

### 3.5. Subgroup analysis

To further analyze the influence of different grouping factors on effect size in these five studies, a subgroup analysis was performed using a fixed-effects model. The subgroup analysis was conducted according to the survey year of publication, country development level, student type, and anxiety type (refer to Table 3). The effect size for the research published in 2021 was −0.208 (95% CI: −0.270, −0.144, *p* < 0.001), while the effect size for the research published in 2021 was −0.218 (95% CI: −0.322, −0.108, *p* < 0.001); no significant regulatory effect was found (*Q* = 0.009, *p* = 0.926). The effect size for the developed country subgroup was −0.148 (95% CI: −0.232, −0.063, *p* = 0.001), while that for the developing country subgroup was −0.257 (95% CI: -−0.325, −0.108, *p* < 0.001); no significant regulatory effect was found (*Q* = 3.728, *p* = 0.054). The effect size for the undergraduate subgroup was −0.190 (95% CI: −0.251, −0.128, *p* < 0.001), while the effect size for the non-undergraduate subgroup was −0.287 (95% CI: −0.397, −0.168, *p* < 0.001); no significant regulatory effect was found (*Q* = 1.986, *p* = 0.159). The effect size for the general anxiety subgroup was −0.234 (95% CI: −0.330, −0.134, *p* < 0.001, while the effect size for the non-general anxiety subgroup was −0.200 (95% CI: −0.264, −0.135, *p* < 0.001); thus, no significant regulatory effect was found (*Q* = 0.231, *p* = 0.631).

**Table 3:** Subgroup analysis of the anxiety and academic performance of university students during the COVID-19 pandemic.

**Subgroup**	**Number of studies**	**Number of samples**	**Heterogeneity**		**Correlation (95%CI)**	***p***	**Total between subgroup analysis**		
			***I** ^2^*	***p***			***Q***	***df***	***p***
**Year of publication**							0.009	1	0.926
2021	3	897	52.72%	0.121	−0.208 (-0.270,−0.144)	<0.001			
2022	2	308	0.00%	0.460	−0.218 (-0.322,−0.108)	<0.001			
**National development level**							3.728	1	0.054
Developed country	2	518	0.00%	0.426	−0.148 (-0.232,−0.063)	0.001			
Developing country	3	687	0.00%	0.803	−0.257 (-0.325,−0.185)	<0.001			
**Student type**							1.986	1	0.159
Undergraduate	3	954	26.57%	0.256	−0.190 (-0.251,−0.128)	<0.001			
Non-undergraduate	2	251	0.00%	0.879	−0.287 (-0.397,−0.168)	<0.001			
**Anxiety type**							0.231	1	0.631
General anxiety	2	359	5.26%	0.001	−0.234 (-0.330,−0.134)	<0.001			
Non-general anxiety	3	846	41.68%	0.050	−0.200 (-0.264,−0.135)	<0.001			

### 3.6. Sensitivity analysis

Each study included in the meta-analysis was individually excluded, and the overall effect size was calculated using a fixed-effects model. There was no significant change in the results before and after exclusion (Table 4), indicating that meta-sensitivity was low, and the results obtained were robust.

**Table 4:** Sensitivity analysis results.

**Excluding**	**Effect size**	**95% CI**	***P***
Chattopadhyay and Sahoo (2022)	−0.205	(−0.261, −0.147)	<0.001
Di Malta et al. (2022)	−0.215	(−0.273, −0.154)	<0.001
Fadhila and Hernawan (2021)	−0.198	(−0.256, −0.139)	<0.001
Kim and Park (2021)	−0.241	(−0.302, −0.178)	<0.001
Noman et al. (2021)	−0.194	(−0.262, −0.125)	<0.001
Overall	−0.211	(−0.264, −0.156)	<0.001

### 3.7. Publication bias

**Figure 3:**
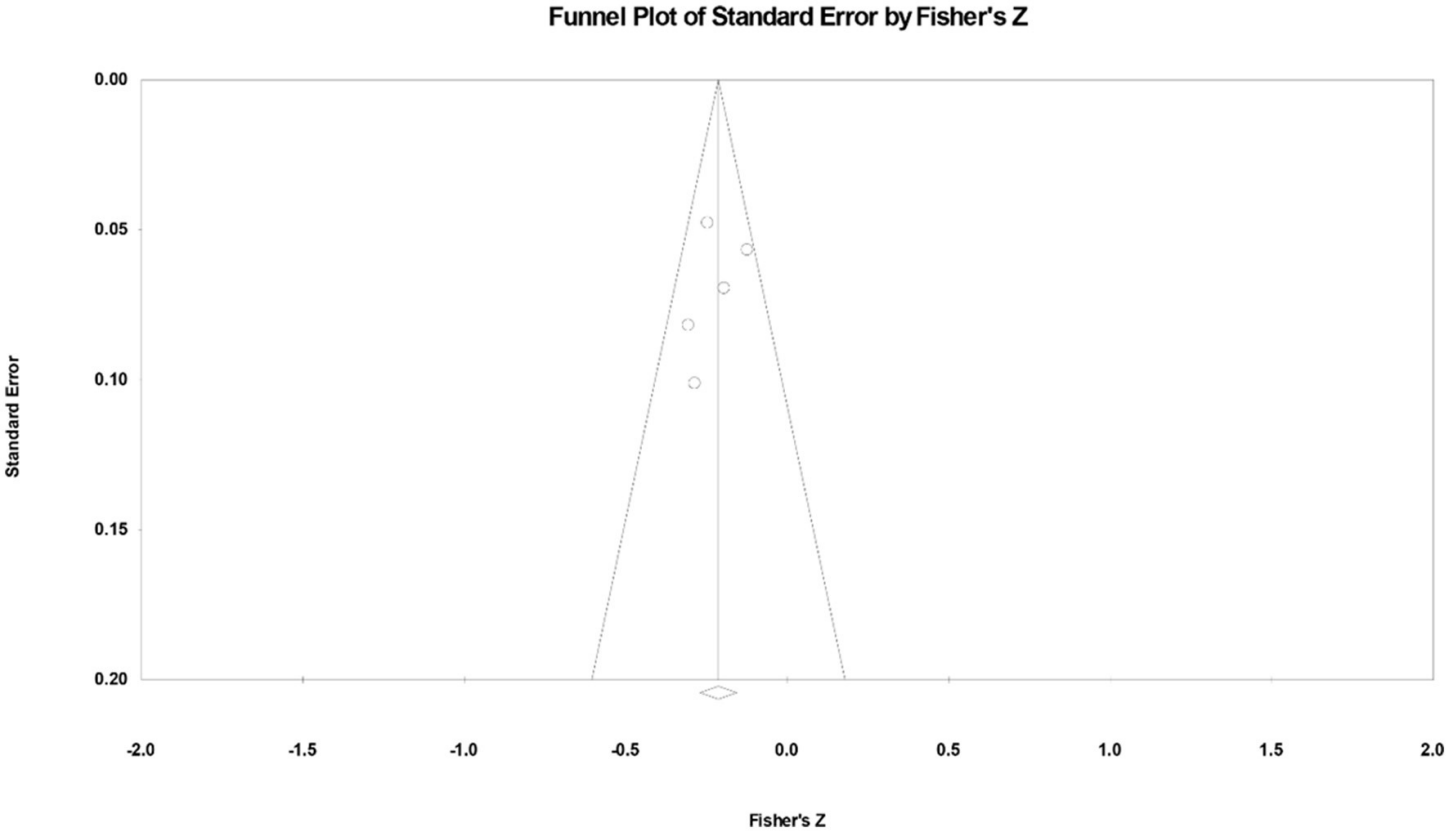
Funnel plot of the anxiety and academic performance of university students during the COVID-19 pandemic. Each point denotes a study.

Using the fixed-effects model, we generated a funnel plot of effect sizes. The funnel diagram was symmetrical around the central axis, whereby the effect sizes of these five studies were evenly distributed on both sides of the total effect (Figure 3). Egger's expression test suggested no evidence of publication bias (*p* > 0.05).

## 4. Discussion

Through the meta-analysis of the correlation between university students' anxiety and academic performance as reported in the five articles, we found that anxiety had a small-to-moderate negative effect on academic performance during the COVID-19 period. The effect size ranged from −0.294 to −0.120, and the mean effect size was −0.211 (*p* < 0.001). The year of publication, country development level, student type, and anxiety type did not exhibit a significant regulatory effect. This meta-analysis was somewhat different from previous studies in this area. We primarily focused on college students, including both undergraduate and postgraduate students. Previous studies did not subdivide college students and did not consider the impact of specific major epidemics. The effect sizes obtained by previous studies were inconsistent, with correlation coefficients ranging from −0.06 to −0.28 (Seipp, 1991; Erzen, 2017; Brumariu et al., 2022). Our effect sizes differ from those reported by Brumariu et al. (2022), who found an effect size r of only −0.03 in the adult student population (18–25 years old). This may be related to the fact that they only considered a clinical anxiety disorder and did not consider the negative academic impact caused by other types of anxiety. In fact, test anxiety and course anxiety are common types of anxiety in students, which have a small-to-moderate negative impact on their academic performance (von der Embse et al., 2018). Effect sizes may be reduced after excluding these anxiety types. Although the effect sizes we report were close to the effect value reported by Erzen (2017) for the university student group (r = −0.27), the current meta-analysis involved a different social environment than that was present in their study. During the pandemic period, more people exhibited anxiety symptoms, and the degree of anxiety was higher. Thus, even if the effect sizes were close, the negative consequences of this anxiety may be very different in the context of a pandemic, potentially leading to larger-scale negative consequences.

In addition to summarizing the effect sizes, we found that negative emotion could be a mechanism underlying this relationship. Studies have reported that negative emotions are an intermediary variable (Noman et al., 2021; Di Malta et al., 2022). Some studies have indicated that COVID-19 has caused university students to feel worried, afraid, and hopeless (Giusti et al., 2021; Ludwig, 2021). The negative emotions of university students caused by the prevalence of COVID-19 may be the fundamental cause of their diminished academic performance. One possible explanation is that different types of anxiety lead university students to experience negative emotions and difficulties in emotional regulation. Cognitive interference theory suggests that the fear of future negative events affects students' normal cognitive and memory processes (Putwain et al., 2010). The attention control model posits that physical anxiety occupies cognitive processing resources and distracts attention from learning (Eysenck et al., 2007). The motivation mediation model proposes that students' emotions have a negative impact on their motivation and goals (Plass and Kalyuga, 2019), indirectly hindering their academic performance. These theoretical models all suggest that university students' anxiety is closely related to diminished academic performance. Previous studies have also found that anxiety may cause difficulty with emotion regulation (Cisler and Olatunji, 2012; Carl et al., 2014; Jazaieri et al., 2014). The anxiety affected the normal emotional regulation of university students and increased their sense of boredom and fear. Students' emotional regulation can mediate the academic output (Genc, 2017). During the pandemic, university students' emotional regulation ability was hindered, which reduced academic performance.

We also found no regulatory effect on the level of economic development of the country where the students were located nor on the type of anxiety of the students, which is consistent with the conclusion of Seipp (1991). The regulatory effect of publication year was also not statistically significant, which may be related to the short publication time interval of these studies and that there was no significant difference in the environment within which the studies were conducted. Previous conclusions regarding the anxiety level of undergraduate and graduate students have been inconsistent (Chrikov et al., 2020; Xiao et al., 2020; Alhasani et al., 2022). Although postgraduate students may have a stronger emotional regulation ability due to their older age, postgraduate students may experience relatively greater difficulties in completing their graduate thesis (Liang et al., 2021). This may be why there was no statistical difference in effect sizes between postgraduate students and undergraduates.

### 4.1. Study strengths and limitations

This study, based on our search of several major research databases, is the first meta-analysis to summarize the relationship between university students' anxiety and learning performance in the context of the COVID-19 pandemic. However, this literature review has some limitations. First, there were few articles on this topic, which limited the number of articles that could be included in the analysis. All of the selected research studies employed a cross-sectional design, which is insufficient for determining the causal relationship. We did not distinguish different forms of anxiety, which may have led to bias in the evaluation of university student's anxiety levels. In addition, this literature review considered only English publications; relevant articles published in other languages may have been omitted.

## 5. Conclusions

This study found for the first time that there was a small-to-moderate negative correlation between university students' anxiety and academic performance during the COVID-19 pandemic. The effects of negative emotions and difficulties with emotion regulation may underlie the influence of university students' anxiety on their academic performance. The year of publication, country development level, student type, and anxiety type had no significant regulatory effect. At present, there are few studies on the impact of different forms of anxiety on academic performance during the COVID-19 pandemic, and it is necessary to further explore this issue. In addition, there is relatively little research on the mechanisms through which university student's anxiety level affects academic performance in this context. Further research is needed to determine the mediating variables and their regulatory role. In the future, it will be necessary to strengthen research on the impact of major pandemics on college student's mental health and academic output, especially given the emerging monkeypox outbreak.

## Data availability statement

The original contributions presented in the study are included in the article/Supplementary material, further inquiries can be directed to the corresponding author.

## Author contributions

YXT was responsible for conceptualization and participated in the methodology design, data extraction, analysis, and original draft writing. WGH participated in data extraction, analysis, review, and editing. Both authors read and approved the final version of the manuscript to be considered for publication.

## References

[B1] Acosta S. Garza T. Hsu H. -Y. Goodson P. (2020). Assessing quality in systematic literature reviews: a study of novice rater training. J. Sage Open. 10:2158244020939530. 10.1177/215824402093953030075189

[B2] Adediwura A. Tayo B. (2007). Perception of teachers' knowledge, attitude and teaching skills as predictor of academic performance in Nigerian secondary schools. Educ. Res. Rev. 2, 165–171.

[B3] Adeoye-Agboola D. I. Evans H. (2015). The relationship between anxiety and academic performance of postgraduate international students in a British University: a cross-sectional quantitative design. Sci. J. Public Health. 3, 331–338. 10.11648/j.sjph.20150303.15

[B4] Alhasani M. Alkhawaji A. Orji R. (2022). Mental health and time management behavior among students during COVID-19 pandemic: towards persuasive technology design. Hum. Behav. Emerg. Technol. 2022, 13. 10.1155/2022/7376748

[B5] Arbona C. Fan W. Phang A. Olvera N. Dios M. (2021). Intolerance of uncertainty, anxiety, and career indecision: a mediation model. J. Career Assess. 29, 699–716. 10.1177/10690727211002564

[B6] Borenstein M. (2009). Effect sizes for continuous data, in The Handbook of Research Synthesis and Meta-Analysis, eds Cooper H. Hedges L. V. Valentine J. C. (New York, NY: Russell Sage Foundation), 221–235.

[B7] Borenstein M. Higgins J. P. T. (2013). Meta-analysis and subgroups. Prev. Sci. 14, 134–143. 10.1007/s11121-013-0377-7 23479191

[B8] Brumariu L. E. Waslin S. M. Gastelle M. Kochendorfer L. B. Kerns K. A. (2022). Anxiety, academic achievement, and academic self-concept: meta-analytic syntheses of their relations across developmental periods. Dev. Psychoathol. 1–17. 10.1017/S0954579422000323 35491696

[B9] Carl J. R. Fairholme C. P. Gallagher M. W. Thompson-Hollands J. Barlow D. H. (2014). The effects of anxiety and depressive symptoms on daily positive emotion regulation. J. Psychopathol. Behav. Assess. 36, 224–236. 10.1007/s10862-013-9387-931045422

[B10] Chattopadhyay M. Sahoo P. K. (2022). Test anxiety of M.Ed. trainees during Covid-19 in relation to their academic achievement. Int. J. Creat. Res. Thought. 10, e60–e72. Available online at: https://ijcrt.org/papers/IJCRT2205451.pdf

[B11] Chrikov I. Soria K. M. Horgos B. Jones-White D. (2020). Undergraduate and Graduate Students' Mental Health During the COVID-19 Pandemic.36802042

[B12] Cisler J. M. Olatunji B. O. (2012). Emotion regulation and anxiety disorders. Curr. Psychiatry. Rep. 14, 182–187. 10.1007/s11920-012-0262-2 22392595 PMC3596813

[B13] Copas J. Shi J. Q. (2000). Meta-analysis, funnel plots and sensitivity analysis. Biostatistics. 1, 247–262. 10.1093/biostatistics/1.3.247 12933507

[B14] Deng J. W. Zhou F. W. Hou W. T. Silver Z. Wong C. Y. Chang O. . (2021). The prevalence of depressive symptoms, anxiety symptoms and sleep disturbance in higher education students during the COVID-19 pandemic: a systematic review and meta-analysis. Psychiatry Res. 301:113863. 10.1016/j.psychres.2021.113863 33984824 PMC9225824

[B15] Di Malta G. Bond J. Conroy D. Smith K. Moller N. (2022). Distance education students' mental health, connectedness and academic performance during COVID-19: a mixed-methods study. Distance Educ. 43, 97–118. 10.1080/01587919.2022.2029352

[B16] Doleck T. Lajoie S. (2018). Social networking and academic performance: a review. Educ. Inf. Technol. 23, 435–465. 10.1007/s10639-017-9612-3

[B17] Erzen E. (2017). The effect of anxiety on student achievement, in The Factors Effecting Student Achievement. Springer. p. 75–94. 10.1007/978-3-319-56083-0_5

[B18] Eysenck M. W. Derakshan N. Santos R. Calvo M. G. (2007). Anxiety and cognitive performance: attentional control theory. Emotion. 7, 336–53. 10.1037/1528-3542.7.2.336 17516812

[B19] Fadhila R. Hernawan B. (2021). Relationship between stress, anxiety, and depression with learning achievement in medical student during online learning in the COVID 19 pandemic era, in Proceedings of ICME 2021 Virtual Conference — Excellence in Health Profession Education Through Globalization & Collaboration, 139–149.

[B20] Fernández-Castillo A. Caurcel M. J. (2015). State test-anxiety, selective attention and concentration in university students. Int. J. Psychol. 50, 265–271. 10.1002/ijop.12092 25104475

[B21] Genc A. (2017). Coping strategies as mediators in the relationship between test anxiety and academic achievement. Genc.Ana. 50, 51–66. 10.2298/PSI160720005G

[B22] Giusti L. Mammarella S. Salza A. Del Vecchio S. Ussorio D. Casacchia M. . (2021). Predictors of academic performance during the covid-19 outbreak: impact of distance education on mental health, social cognition and memory abilities in an Italian university student sample. BMC. Psychol. 9, 142. 10.1186/s40359-021-00649-9 34526153 PMC8441245

[B23] Gusenbauer M. Haddaway N. R. (2020). Which academic search systems are suitable for systematic reviews or meta-analyses? evaluating retrieval qualities of Google Scholar, PubMed, and 26 other resources. Res. Synthesis Method. 11, 181–217. 10.1002/jrsm.1378 31614060 PMC7079055

[B24] Hamzah F. Mat K. C. Bhagat V. Mahyiddin N. S. (2018). Test anxiety and its impact on first year university students and the over view of mind and body intervention to enhance coping skills in facing exams. Res. J. Pharm. Technol. 11, 2220–2228. 10.5958/0974-360X.2018.00411.0

[B25] Huarcaya-Victoria J. Elera-Fitzcarrald C. Crisol-Deza D. Villanueva-Zúñiga L. Pacherres A. Torres A. . (2021). Factors associated with mental health in Peruvian medical students during the COVID-19 pandemic: a multicentre quantitative study. Rev. Colomb. Psiquiatr. 10.1016/j.rcp.2021.06.002 34275600 PMC9186139

[B26] Huedo-Medina T. B. Sánchez-Meca J. Marín-Martínez F. Botella J. (2006). Assessing heterogeneity in meta-analysis: Q statistic or I^2^ index? Psychol. Methods. 11, 193–206. 10.1037/1082-989X.11.2.193 16784338

[B27] Islam M. A. Barna S. D. Raihan H. Khan M. N. A. Hossain M. T. (2020). Depression and anxiety among university students during the COVID-19 pandemic in Bangladesh: a web-based cross-sectional survey. PLoS ONE. 15:e0238162. 10.1371/journal.pone.0238162 32845928 PMC7449469

[B28] Islam S. Akter R. Sikder T. Griffiths M. D. (2020). Prevalence and factors associated with depression and anxiety among first-year university students in Bangladesh: a cross-sectional study. Int. J. Ment. Health. Addict. 20, 1289–1302. 10.1007/s11469-020-00242-y

[B29] Jazaieri H. Morrison A. S. Goldin P. R. Gross J. J. (2014). The role of emotion and emotion regulation in social anxiety disorder. Curr. Psychiatry Rep. 17, 531. 10.1007/s11920-014-0531-3 25413637

[B30] Jones P. J. Park S. Y. Lefevor G. T. (2018). Contemporary college student anxiety: the role of academic distress, financial stress, and support. J. Coll. Counsell. 21, 252–264. 10.1002/jocc.12107

[B31] Karjanto N. Yong S. T. (2013). Test anxiety in mathematics among early undergraduate students in a British university in Malaysia. Eur. J. Eng. Educ. 38, 11–37. 10.1080/03043797.2012.742867

[B32] Kim S. H. Park S. (2021). Influence of learning flow and distance e-learning satisfaction on learning outcomes and the moderated mediation effect of social-evaluative anxiety in nursing college students during the COVID-19 pandemic: A cross-sectional study. Nurs. Educ. Pract. 56, 103197. 10.1016/j.nepr.2021.103197 34537671 PMC8419785

[B33] Leppavirta J. (2011). The impact of mathematics anxiety on the performance of students of electromagnetics. J. Engin. Educ. 100, 424–443. 10.1002/j.2168-9830.2011.tb00021.x

[B34] Li W. Zhao Z. Chen D. Peng Y. Lu Z. (2022). Prevalence and associated factors of depression and anxiety symptoms among college students: a systematic review and meta-analysis. J. Child Psychol. Psychiatry 63, 1222–1230. 10.1111/jcpp.13606 35297041

[B35] Liang Z. Zeng Q. Zhang M. Luo H. Huang S. Li J. . (2021). How course support and academic support impact on Chinese graduate students during the COVID-19: the multiple mediating roles of thesis writing and anxiety. Int. J. Environ. Res. Public Health. 19, 265. 10.3390/ijerph19010265 35010522 PMC8751128

[B36] Lin L. Chu H. J. B. (2018). Quantifying publication bias in meta-analysis. Biometrics. 74, 785–794. 10.1111/biom.12817 29141096 PMC5953768

[B37] Liu M. H. Xu H. L. (2021). Testing effects of foreign language listening anxiety on chinese university students' english listening test performance. Front. Psychol. 12:701926. 10.3389/fpsyg.2021.701926 34322069 PMC8310933

[B38] Lozano Blasco R. Latorre Cosculluela C. Quílez Robres A. (2020). Social network addiction and its impact on anxiety level among university students. Sustainability. 12, 5397. 10.3390/su12135397

[B39] Ludwig J. (2021). Poor performance in undergraduate math: Can we blame it on COVID-19 despair. Int. J. Innov. Sci. Math. 9, 31–40.

[B40] MacCann C. Jiang Y. Brown L. E. Double K. S. Bucich M. Minbashian A. (2020). Emotional intelligence predicts academic performance: a meta-analysis. Psychol. Bull. 146, 150. 10.1037/bul0000219 31829667

[B41] McNaughton N. (2018). What do you mean ‘anxiety'? developing the first anxiety syndrome biomarker. J. R. Soc. N. Z. 48, 177–190. 10.1080/03036758.2017.1358184

[B42] Moher D. Shamseer L. Clarke M. Ghersi D. Liberati A. Petticrew M. . (2015). Preferred reporting items for systematic review and meta-analysis protocols (PRISMA-P) 2015 statement. Syst. Rev. 4, 1–9. 10.1186/2046-4053-4-1 25554246 PMC4320440

[B43] Noman M. Kaur A. Nafees N. (2021). Covid-19 fallout: Interplay between stressors and support on academic functioning of Malaysian university students. Child. Youth. Serv. Rev. 125:106001. 10.1016/j.childyouth.2021.106001 35990216 PMC9375173

[B44] Plass J. L. Kalyuga S. (2019). Four ways of considering emotion in cognitive load theory. Educ. Psychol. Rev. 31, 339–359. 10.1007/s10648-019-09473-5

[B45] Putwain D. W. Connors L. Symes W. (2010). Do cognitive distortions mediate the test anxiety–examination performance relationship? Educ. Psychol. 30, 11–26. 10.1080/01443410903328866

[B46] Richardson M. Abraham C. Bond R. (2012). Psychological correlates of university students' academic performance: a systematic review and meta-analysis. Psychol. Bull. 138, 353. 10.1037/a0026838 22352812

[B47] Sakin Ozen N. Ercan I. Irgil E. Sigirli D. (2010). Anxiety prevalence and affecting factors among university students. Asia Pac. J. Public Health 22, 127–133. 10.1177/1010539509352803 20032042

[B48] Seipp B. J. A. (1991). Anxiety and academic performance: a meta-analysis of findings. Anxiety. Res. 4, 27–41. 10.1080/08917779108248762

[B49] Singh S. Malik S. Singh P. (2016). Research paper factors affecting academic performance of students. Indian J. Res. 5, 176–178.

[B50] Spitzer R. L. Kroenke K. Williams J. B. W. Lowe B. (2006). A brief measure for assessing generalized anxiety disorder - the GAD-7. Arch. Intern. Med. 166, 1092–1097. 10.1001/archinte.166.10.1092 16717171

[B51] Sterne J. A. Sutton A. J. Ioannidis J. P. Terrin N. Jones D. R. Lau J. . (2011). Recommendations for examining and interpreting funnel plot asymmetry in meta-analyses of randomised controlled trials. BMJ. 343:d4002. 10.1136/bmj.d4002 21784880

[B52] Sustarsic M. Zhang J. (2022). Navigating through uncertainty in the era of COVID-19: Experiences of international graduate students in the United States. J. Int. Stud. 12, 61–80. 10.32674/jis.v12i1.3305

[B53] Talsma K. Schüz B. Schwarzer R. Norris K. (2018). I believe, therefore I achieve (and vice versa): a meta-analytic cross-lagged panel analysis of self-efficacy and academic performance. Learn. Individ. Differ. 61, 136–150. 10.1016/j.lindif.2017.11.015

[B54] Visser M. Law-van Wyk E. (2021). University students' mental health and emotional wellbeing during the COVID-19 pandemic and ensuing lockdown. S. Afr. J. Psychol. 51, 229–243. 10.1177/00812463211012219 PMC8144883 38603064

[B55] Vitasari P. Wahab M. N. A. Othman A. Awang M. G. (2010). A research for identifying study anxiety sources among university students. Int. Educ. Stud. 3, 189–196. 10.5539/ies.v3n2p189

[B56] von der Embse N. Jester D. Roy D. Post J. (2018). Test anxiety effects, predictors, and correlates: a 30-year meta-analytic review. J. Affect. Disord. 227, 483–493. 10.1016/j.jad.2017.11.048 29156362

[B57] Walters K. S. Bulmer S. M. Troiano P. F. Obiaka U. Bonhomme R. (2018). Substance use, anxiety, and depressive symptoms among college students. J. Child Adolesc. Subst. Abuse 27, 103–111. 10.1080/1067828X.2017.1420507

[B58] Wang X. Chen H. Liu L. Liu Y. Zhang N. Sun Z. . (2020). Anxiety and sleep problems of college students during the outbreak of COVID-19. Front. Psychiatry. 11:588693. 10.3389/fpsyt.2020.588693 33329134 PMC7719633

[B59] Xiao H. Shu W. Li M. Li Z. Tao F. Wu X. . (2020). Social distancing among medical students during the 2019 coronavirus disease pandemic in China: disease awareness, anxiety disorder, depression, and behavioral activities. Int. J. Environ. Res. Public Health. 17:5047. 10.3390/ijerph17145047 32674285 PMC7399842

[B60] Zhang C. Shi L. Tian T. Zhou Z. Peng X. Shen Y. . (2022). Associations between academic stress and depressive symptoms mediated by anxiety symptoms and hopelessness among Chinese college students. Psychol. Res. Behav. Manag. 15, 547–556. 10.2147/PRBM.S353778 35282002 PMC8906854

[B61] Zhang J. Zhao N. Kong Q. P. (2019). The relationship between math anxiety and math performance: A meta-analytic investigation. Front. Psychol. 10:1613. 10.3389/fpsyg.2019.01613 31447719 PMC6692457

